# Rare Genetic Blood Disease Modeling in Zebrafish

**DOI:** 10.3389/fgene.2018.00348

**Published:** 2018-08-31

**Authors:** Alberto Rissone, Shawn M. Burgess

**Affiliations:** Translational and Functional Genomics Branch, National Human Genome Research Institute, National Institutes of Health, Bethesda, MD, United States

**Keywords:** zebrafish, hematopoiesis, modeling human diseases, SCID, blood, genome editing

## Abstract

Hematopoiesis results in the correct formation of all the different blood cell types. In mammals, it starts from specific hematopoietic stem and precursor cells residing in the bone marrow. Mature blood cells are responsible for supplying oxygen to every cell of the organism and for the protection against pathogens. Therefore, inherited or *de novo* genetic mutations affecting blood cell formation or the regulation of their activity are responsible for numerous diseases including anemia, immunodeficiency, autoimmunity, hyper- or hypo-inflammation, and cancer. By definition, an animal disease model is an analogous version of a specific clinical condition developed by researchers to gain information about its pathophysiology. Among all the model species used in comparative medicine, mice continue to be the most common and accepted model for biomedical research. However, because of the complexity of human diseases and the intrinsic differences between humans and other species, the use of several models (possibly in distinct species) can often be more helpful and informative than the use of a single model. In recent decades, the zebrafish (*Danio rerio*) has become increasingly popular among researchers, because it represents an inexpensive alternative compared to mammalian models, such as mice. Numerous advantages make it an excellent animal model to be used in genetic studies and in particular in modeling human blood diseases. Comparing zebrafish hematopoiesis to mammals, it is highly conserved with few, significant differences. In addition, the zebrafish model has a high-quality, complete genomic sequence available that shows a high level of evolutionary conservation with the human genome, empowering genetic and genomic approaches. Moreover, the external fertilization, the high fecundity and the transparency of their embryos facilitate rapid, *in vivo* analysis of phenotypes. In addition, the ability to manipulate its genome using the last genome editing technologies, provides powerful tools for developing new disease models and understanding the pathophysiology of human disorders. This review provides an overview of the different approaches and techniques that can be used to model genetic diseases in zebrafish, discussing how this animal model has contributed to the understanding of genetic diseases, with a specific focus on the blood disorders.

## Introduction

Genetic diseases can be both inherited and acquired. In particular, most of the inherited diseases belong to the category called “orphan diseases” (Strynatka et al., 2018). The term orphan disease can refer to two different types: common diseases neglected by doctors or rare diseases with a variable incidence in the population (Aronson, 2006). Although for different reasons, both have in common minimal scientific research about their genetic causes and molecular mechanisms and a lack of investments by pharmaceutical sector to develop new treatments (Strynatka et al., 2018). The definition of rare disease in not universal and depends on the country. In the United States, for example, a disease is considered rare when affecting fewer than 1 person in 200,000, but in Japan and Australia the numbers are very different: 1/50,000 and 1/2,000, respectively (Lavandeira, 2002).

With the advent of next-generation sequencing technologies and a progressive reduction in sequencing costs, we will begin to see a dramatic increase in the identification of the genes responsible for human genetic disorders. Model organisms played a pivotal role in genotype-to-phenotype studies, in particular when the association is unclear (Strynatka et al., 2018). However, with an estimated total number of Mendelian genetic diseases between 7,000 and 15,000 (Boycott et al., 2013) and only ∼1,500 drugs approved by FDA, most of the genetic diseases still have no effective treatment, indicating a constant need for new experimental animal models.

### Animal Models to Study Genetic Diseases

Animal models are fundamental tools in biomedical research because they can fill the gap between basic science and the treatment of human diseases (Zon, 2016). Several different animal models can be used to study the gene function providing new insight into pathophysiology of human disorders (Bier and McGinnis, 2004). Simple models such as *Saccharomyces cerevisiae* (Foury, 1997) and *Dictyostelium discoideum* (Firtel and Chung, 2000; Chung et al., 2001) proved to be very helpful in elucidating the basic mechanisms of eukaryotic cell function, such as the regulation of the cell cycle, the mechanisms of DNA damage and repair, metabolism, and cell signaling. Similarly, invertebrates like *Caenorhabditis elegans* (Aboobaker and Blaxter, 2000; Culetto and Sattelle, 2000) and *Drosophila melanogaster* (Bernards and Hariharan, 2001; Reiter et al., 2001; Chien et al., 2002) represent outstanding models to study genes involved in more complex body plans (Bier and McGinnis, 2004). However, their very high evolutionary distance with a low rate of sequence conservation compared to vertebrates and the huge difference in their anatomy and physiology, limit their use in studying vertebrate-specific embryonic development and in directly modeling human diseases.

Traditionally, among mammals *Mus musculus* (mouse) and *Rattus norvegicus* (rat) are the species most commonly used as a vertebrate model organisms. Particularly the mouse with its small size, genomic resources, genetic tractability, and anatomic and physiologic conservation with humans, elected it as the favored species to model human genetic disorders. Although in the past, mouse models were usually generated using homologous recombination methods in embryonic stem cells (ESCs) it was a laborious, time consuming and not so efficient approach. With the advent of the new genome-editing techniques the overall process has been speed-up and today the generation of new mouse models require just few weeks, instead of the previous 1–2 years (Ott de Bruin et al., 2015). However, the maintenance of large mouse colonies is still expensive reducing its use in large-scale genetic screens and phenotyping studies. In addition, because of the complexity of human diseases and the intrinsic differences between humans and other species, it is often the case that some aspects of the model organisms physiology makes it a poor model for a specific disease, and so multiple model organisms are needed. Based on several features described in detail below, zebrafish represents a good compromise for modeling human diseases, filling the gap between the invertebrate and mammalian model systems.

### Zebrafish as an Animal Model

The zebrafish (*Danio rerio*) is a tropical freshwater fish from South-East Asia which has in recent decades gained popularity in the research community. Zebrafish popularity began at the end of the last century (1970s–1980s), when they became a new genetic model for developmental biologists. However, because of the numerous advantages that zebrafish offer, it has rapidly become popular in the study of human disease.

Zebrafish belong to the teleost clade, also known as “bony” fish. The eggs are externally fertilized which allows for simple experimental manipulation of the embryos, and each mating produces a high number (usually >100) of embryos. The embryo development is very fast compared to other vertebrate models such as mice, and a few days after the hatching (48–72 h post fertilization) zebrafish embryos already show all the major organs of the adult animals. Notably, the anatomy and physiology of most of the zebrafish organs are very similar to those of mammals and in terms of hematopoiesis, Teleosts have all the different hematopoietic cellular elements found in mammals (i.e., erythroid, myeloid and lymphoid lineages).

Although under normal conditions zebrafish embryos are not completely transparent, they may be treated with 1-phenyl 2-thiourea (PTU) at ∼24 h post fertilization (hpf) which will inhibit melanin formation resulting in almost transparent embryos that will continue to remain in this condition as long as the PTU treatment is continued (Karlsson et al., 2001). Alternatively, numerous genetic pigmentation mutants with different levels of transparency are available and can permit *in vivo* imaging from the embryo phases to adulthood (White et al., 2008).

The zebrafish is well suited for molecular and genetic analysis of temporal and spatial gene expression using whole mount *in situ* hybridization (WISH) (Thisse and Thisse, 2008); moreover, a very long list of transgenic lines (including inducible models) are publicly available that allows study of tissue and organ development *in vivo* and in real-time during all the phases of embryo development (Kondrychyn et al., 2011; Ruzicka et al., 2015). For a comprehensive list of transgenic lines helpful in studying zebrafish hematopoiesis see Gore et al. (2018).

Thousands of mutations obtained using large scale mutagenesis screens are available and moreover new mutations can be easily introduced in zebrafish genome using the most recent techniques of site-specific genome editing such as the Clustered Regularly Interspaced Short Palindromic Repeat/CRISPR associated protein 9 (CRISPR/Cas9). The zebrafish genome has been fully sequenced and high-quality assemblies are publically available (Howe et al., 2013). Genomic analysis shows that there is a high degree of sequence conservation and synteny between the zebrafish and human genomes. Zebrafish, especially during its embryonic stages, proved to be very suitable for medium- to large-throughput drug screening, because it is possible to add the different compounds directly into the embryo medium. Moreover, usually zebrafish bioassays are cheaper and faster than the comparable mouse assays. Finally, maintenance costs of zebrafish model are lower than those for mammals. While this review focuses on mutational analysis in early embryos, adult zebrafish are increasingly being used to study some blood diseases as well, particularly blood cancers (Langenau et al., 2003; Alghisi et al., 2013).

Like any other animal model and despite its numerous advantages and unique features, the zebrafish model system is not devoid of disadvantages and/or limitations. One of major limitations is the teleost-specific genome duplication. This event occurred ∼400 millions of years ago and corresponded to a complete duplication of teleost genome (Meyer and Van de Peer, 2005). After the duplication event, the majority of the duplicated genes were lost or became pseudogenes. However, roughly 20% of the genes maintained two functional copies in the genome. As a results, zebrafish and other teleost species have a higher number of protein coding genes (∼26,000) compared to other vertebrates like human, mouse or chicken (∼21,000) (Howe et al., 2013). This information must be taken in consideration during reverse genetic analysis of the duplicated genes as a result of compensatory effects, most of the cases knocking out one copy of the gene is not enough to mimic the effects of a null allele. Another important aspect to take into consideration is that, after gene duplication, each duplicate can functionally diverge from each other through sub-functionalization and/or neo-functionalization events (Ohno, 1970; Postlethwait et al., 2004; Rastogi and Liberles, 2005). Although the modern genome-editing technologies allow the targeting of multiple genes at the same time, somewhat overcoming the problem duplicate genes, neo-functionalization events could have partially changed the gene’s function. These phenomena could be responsible for discrepant functional outcomes among models in different species and potentially could limit the use of zebrafish in modeling human diseases. Unfortunately, it is currently not possible to determine *a priori* whether this would be an issue for any given gene duplication.

### Zebrafish as a Tool to Study Human Diseases

The recent advent in the zebrafish field of targeted genome editing techniques, such as ZFN, TALEN, and in particular CRISPR/Cas9, has opened up the model to reproduce human pathological conditions of known disease-related genes and to study their effects *in vivo*, with the ultimate goal of identifying new therapeutic targets (Detrich et al., 1999; Langheinrich, 2003; Santoriello and Zon, 2012). Although historically the first successful attempts to use zebrafish for genetic studies were represented by forward genetic approaches using chemical or insertional mutagenesis techniques (Haffter and Nusslein-Volhard, 1996; Golling et al., 2002; Varshney et al., 2013), later on, thanks to the development of knockdown and targeted genome editing techniques, this model system proved that it could be efficiently used in reverse genetic approaches as well.

#### Knockdown Approach to Study Gene Function

In zebrafish, with the exception of limited cases where RNAi has been used to knockdown specific targets (Oates et al., 2000), the knockdown approach has been performed through the use of morpholinos (MOs) (Nasevicius and Ekker, 2000). Because of their ease of use, MOs represented the first and they are still today one of the most popular approaches to perform reverse genetic analysis in zebrafish (Eisen and Smith, 2008; Bill et al., 2009; Timme-Laragy et al., 2012; Stainier et al., 2017). MOs are modified antisense oligonucleotides (ASOs) that are manually microinjected in the embryos at the first stages of development (1–4 cells maximum). MO oligonucleotides are very stable because they are not targeted by nuclease enzymes and they do not act through an RNaseH mechanism, as in the case of RNA interference (RNAi) technology (Eisen and Smith, 2008). Instead, through the binding to their RNA targets (pre-mRNA or mRNA), MOs induce a transient dose-dependent knockdown effect in the injected embryos (morphants). During embryonic development, MOs concentration is gradually reduced in the cells due to cell divisions and therefore they do not typically stay effective beyond 3–5 days post fertilization (dpf) (Timme-Laragy et al., 2012).

Usually, low doses of MOs are well tolerated by zebrafish embryos, allowing the targeting of more than a single transcript at the same time to study synergistic effects of multiple knockdowns (Rissone et al., 2012). While the report of potential off-target effects mediated by the *tp53* activation following MOs injection (Robu et al., 2007) raised several criticisms to the validity of some results obtained with them (Blum et al., 2015; Kok et al., 2015; Stainier et al., 2015), they have been extensively used to study gene function during embryo development and to confirm the role of candidate genes involved in human diseases. Usually, if used with full knowledge of the potential risks and limitations and with all the essential controls (Eisen and Smith, 2008; Stainier et al., 2015, 2017), MOs represents a good starting point to infer gene function in a fast and inexpensive way and, at least in one case, it has been shown that MOs action can prevent the genetic compensatory effects induced in some mutant animals (Rossi et al., 2015).

Recently, an alternative knockdown approach requiring the injection of RNA–DNA hybrid ASOs (also known as gapmers) has been used to overcome some of the limitations of MOs (Pauli et al., 2015): first, each molecule of a steric-blocking MO will only bind a single target RNA transcript; second, translational MOs, designed to block the ATG codon of mRNAs, do not induce the degradation of the target transcripts impeding the quantification of their knockdown efficiency. In contrast, gapmer ASOs contains a central DNA region, which triggers an RNAse H-mediated degradation of the target RNAs. The molecules also have flanking 2′-modified nucleosides at both ends to protect them from exonucleases activity and to increase the affinity for the targets (Evers et al., 2015). While they have already been used in cell culture (Dimitrova et al., 2014) and other species (Heasman et al., 1994; Zhang et al., 1998; Wheeler et al., 2012), gapmers-mediated knockown represents a relatively unexplored approach in zebrafish. Pauli et al. (2015) tested the feasibility of this approach in zebrafish targeting ∼20 protein-coding and non-coding transcripts with known embryonic loss-of-functions phenotypes and showing that gapmers can represent an effective RNA knockdown alternative. Although representing a promising tool, more studies are required to further confirm their potential utility.

Finally, given the continuous reduction of the costs required to create mutant alleles in zebrafish (Varshney et al., 2015b), in the future the studies involving the use of MOs or other knockown techniques could and should include a comparison of the phenotypes observed in bona fide genetic mutants as a control (Stainier et al., 2017).

#### Zebrafish Genome Editing Tools

In zebrafish, large-scale genetic screens using random mutagenesis were successfully introduced at the end of the 1990s (Haffter and Nusslein-Volhard, 1996; Haffter et al., 1996). These forward genetic techniques proved to be very helpful in identifying mutants presenting phenotypes typical of several human disorders (North and Zon, 2003; Amsterdam and Hopkins, 2006; Bradford et al., 2017; Howe et al., 2017). However, these approaches were not devoid of limitations; especially in the amount of efforts required to isolate each mutation by positional cloning and they were gradually replaced by the use of reverse genetic approaches. In particular, the advent of the TILLING (or Targeting Induced Local Lesions in Genome) system in early 2000s (Wienholds et al., 2002) allowed the researchers to screen for mutations in specific genes of interest. Later, new and more efficient tools for targeted mutagenesis were developed and they were quickly adopted by the zebrafish community (Doyon et al., 2008; Meng et al., 2008; Huang et al., 2011; Bedell et al., 2012). Briefly, all the major genome editing techniques are based on the coupling of DNA-binding domains or guide RNA molecules to proteins with nuclease activity used to induce double-stranded breaks (DSB) in the target genomic regions. Then inductions of DSBs prompts the activity of two different cellular DNA repair mechanisms: (a) the most common, but highly error–prone non-homologous end joining (NHEJ) and (b) the homology-directed repair (HDR) which is rarer *in vivo* and requires a template DNA to repair the DSB (Symington and Gautier, 2011). The HDR mechanism seems to be particularly challenging in zebrafish and so far, despite numerous efforts of the community to optimize the mutagenic protocol in order to increase its frequency in zebrafish, very few successful cases are described in literature (Hruscha et al., 2013; Hwang et al., 2013a; Auer et al., 2014; Irion et al., 2014; Kimura et al., 2014; Hisano et al., 2015; Li et al., 2015; Armstrong et al., 2016; Hoshijima et al., 2016; Zhang et al., 2016; Moreno-Mateos et al., 2017; Zhang et al., 2018). In contrast, in zebrafish the NHEJ repair mechanism works very efficiently and usually it is associated with loss/gain of small fragments of genomic DNA in the range of 1 bp to ∼40 bps. Selecting frame-shift mutations introduced by NHEJ potentially impairs the structure and/or the functionality of the targeted protein.

The first examples of zebrafish mutants obtained with Zinc Finger Nucleases (ZFNs) and Transcription Activator-Like Effector Nucleases (TALENs) were published in the late 2000s and 2011, respectively (Doyon et al., 2008; Meng et al., 2008; Huang et al., 2011; Sander et al., 2011). They both allowed an easy targeting and recovery of the different mutations introduced in the specific genomic regions, although their major limitations resided in their still high costs and in the efforts necessary to develop the modular DNA-binding motifs responsible for the sequence specificity.

The recent advent of CRISPR/Cas9 system moved the versatility and affordability of the genome editing in zebrafish to a new level, allowing the targeting of multiple regions at the same time (multiplexing) with a consistent reduction of the costs (Hwang et al., 2013b; Jao et al., 2013; Varshney et al., 2015a). In the CRISPR/Cas9 system, the sequence specificity is obtained using an ∼20 base pairs long guide RNA (gRNA), while the DNA double-strand cleavage activity is attained using the Cas9 endonuclease activity. Compared to ZFNs and TALENs, CRISPR/Cas9 system has a similar or better efficiency in targeting genomic DNA, with a higher versatility and simplicity of design. The only limitation in the target design consist in the presence of a protospacer adjacent motif (PAM) directly upstream the target region bound by the gRNA. The PAM sequence depends on the Cas9 protein used, but in most of the cases is the nucleotide sequence NGG (Varshney et al., 2015b). Based on the biological target system used (cells or animal embryos) the gRNA sequence is delivered by transfection of specific gRNA-containing vectors or direct injection of gRNAs generated by *in vitro* transcription. In zebrafish, its intrinsic features such as the external fertilization and the easy manipulation of the embryos, allow the direct co-injection of Cas9 mRNA (or Cas9 protein) and the gRNAs into the embryos during the earliest stages of development (similarly to MOs). One further advantage of the zebrafish embryos is that they can easily tolerate multiple gRNAs at the same time, making possible a multiplexing approach that dramatically increases its versatility. It becomes possible to target multiple genes at the same time or to use specific gRNA directed against different regions of a single gene to maximize the number of mutated allele or to induce the deletion of a large genomic region based on the specific needs of the researchers. Another benefit of a multiplexing approach in zebrafish consists in overcoming the potential compensatory effects due to the duplicated genes, which clearly represents a problem for genetic analysis in this animal model. In contrast, an obvious downside of a multiplexing approach is represented by the increased probability of off-target activity, which seems to be relatively rare, but detectable (Varshney et al., 2015a). Recently, in order to maximize the specificity of the CRISPR/Cas9 system, different variants of the Cas9 enzyme (Cas9n and Cas9/FokI) were successfully developed and proved to work very well in limiting nuclease activity to specific genomic sites, although reducing at the same time the overall multiplex potential of the system (Ran et al., 2013; Tsai et al., 2014). Many human diseases are due to mutations predicted to cause single or multiple amino acid substitutions that partially inhibit gene activity, instead of completely impairing protein function or mRNA stability. Therefore, in order to better mimicking the mutations found in human patients, the ability to introduce in an animal substitution genetic mutations (in contrast to null alleles) represents one of the current challenges of the genomic editing era. Recently, a new genome editing technique called “base editing” has been developed and tested in mammalian cells and different model species (Komor et al., 2016; Kim et al., 2017). Through the fusion of a cytidine deaminase enzyme to the N-terminal region of a Cas9 nickase (nCas9) protein, this new technology allows direct conversion of one single base in a programmable way bypassing the DSBs. Recently, Zhang et al. (2017) adapted a similar approach to work efficiently in zebrafish, further increasing its versatility as animal model in modeling human diseases.

### Zebrafish Hematopoiesis

Usually, during both zebrafish and mammal embryo development, hematopoiesis is obtained in three distinctive, but partially overlapping, processes termed “hematopoietic waves” (Ciau-Uitz et al., 2014). For correct hematopoietic development, both their timing and embryonic localization need to be strictly regulated (for a comprehensive list of the genes expressed and involved in the different phases of zebrafish hematopoiesis see Gore et al., 2018). Based on the type of blood cells originated, the three major hematopoietic waves are distinguished in primitive, prodefinitive (or intermediate) and definitive. In mammals, during the first two hematopoietic waves, red blood cells and macrophages, and erythroid-myeloid precursors are generated extra-embryonically in the yolk sac blood island (Ciau-Uitz et al., 2014). In contrast, the definitive hematopoiesis produces all the major hematopoietic cell types (erythroid, myeloid, and lymphoid) through the creation of hematopoietic stem and precursor cells (HSPCs).

Over the years, several reports pointed out the utility of the zebrafish model to study the different aspects of vertebrate hematopoiesis (Gore et al., 2018). Despite the >400 million years of evolutionary distance (Postlethwait et al., 1999) and the different embryonic territories involved (Ciau-Uitz et al., 2014), zebrafish and mammal species share the same key genetic regulation and mechanisms (Sood and Liu, 2012; Avagyan and Zon, 2016; Gore et al., 2018).

#### Zebrafish Primitive Hematopoiesis

In zebrafish, primitive hematopoiesis starts around 11 h post fertilization (hpf) during somitogenesis (Davidson and Zon, 2004). Specific cells inside the anterior and posterior lateral mesoderm (ALM and PLM, respectively) start expressing endothelial and hematopoietic markers generating different populations of vascular and hematopoietic precursors. Later, the ALM gives rise to the rostral blood island (RBI) region. The cells in the ALM/RBI region generate primitive myeloid precursors which eventually differentiate into macrophages and neutrophils (Herbomel et al., 1999). Specifically, primitive myeloid precursors start to express the transcription factor *pu.1* and then they leave the RBI spreading on the yolk sac (Galloway et al., 2005; Rhodes et al., 2005; Monteiro et al., 2011). Following *pu.1* activation, different myeloid markers start to be expressed in these migrating cells and finally, with the expression of *irf8*, *cebpa*, and *cebp1* genes, these precursors begin to assume a more restricted myeloid identity (Li et al., 2011; Jin et al., 2012, 2016; Mommaerts et al., 2014; Dai et al., 2016). Precursor cells from RBI are also responsible for the generation of mast cells (Dobson et al., 2008) and microglia, which will eventually colonize the brain (Xu et al., 2015, 2016). In contrast, the future hematopoietic precursors that reside in the PLM migrate, converging to the midline of the embryo body and forming the intermediate cell mass (ICM). The ICM occupies the space between the yolk extension and the notochord and it extends throughout the trunk of the embryos to the end of the yolk extension (Ciau-Uitz et al., 2014). Although the hematopoietic precursors forming the ICM region during development express both erythroid and myeloid markers (such as *gata1a*, and *spi1b* or *mpx*, respectively) this posterior first hematopoietic wave seems to generate mostly primitive erythrocytes that enter the circulation at ∼24 hpf, when the zebrafish heart starts beating (Berman et al., 2003). Primitive erythroid and myeloid cells are then gradually replaced by blood cells produced during the following hematopoietic waves.

#### Zebrafish Prodefinitive Hematopoiesis

Around 24 hpf, the zebrafish embryos switch from the primitive to the definitive hematopoiesis. This transition occurs concurrently in two different regions of the embryo: the posterior blood island (PBI) and the ventral wall of the dorsal aorta (VDA). From the VDA originates the precursors of HSPCs; while in the PBI, which is the most caudal region of the ICM right after the end of the yolk extension, only erythroid-myeloid progenitors (EMP) begin to differentiate (Bertrand et al., 2007). Because they do not derive from HSPCs, the hematopoietic potential of EMPs is limited to the erythroid and myeloid lineages (Gore et al., 2018). However, a very recent temporally–spatially resolved fate-mapping analysis (Tian et al., 2017) showed that the ventral endothelium in the VDA and PBI regions, gives rise to a transient wave of T lymphopoiesis which does not require HSPCs. Notably, the generated T-cells are mostly CD4 Tαβ cells and are temporally limited to the larval stages of development (Tian et al., 2017).

#### Zebrafish Definitive and Adult Hematopoiesis

The formation of HSPCs is the characteristic step of definitive hematopoiesis. Compared to previous hematopoietic progenitors generated during primitive hematopoiesis, HSPCs are self-renewable pluripotent stem cells responsible for the formation of all the hematopoietic lineages during the zebrafish development and the adult phase. In zebrafish the definitive hematopoiesis starts around ∼24 hpf with the formation of the first *runx1* and then *cmyb* positive cells in a specific region of the trunk of the embryos, the VDA which functionally corresponds to the aorta-gonad-mesonephros (AGM) region in mammals. In the VDA, a subset of endothelial cells starts to differentiate in HSPCs and they eventually bud from the aorta and colonize the region between the aorta and the posterior cardinal vein (PCV) (Bertrand et al., 2010; Kissa and Herbomel, 2010). This process is usually indicated as endothelial-hematopoietic transition (EHT) (Kissa and Herbomel, 2010; Bresciani et al., 2014). Then, HSPCs enter the circulation through the PCV and move caudally to colonize by 2 dpf the so called caudal hematopoietic tissue (CHT) which consists of the region where the vascular plexus connecting the dorsal aorta and the cardinal vein resides (Murayama et al., 2006; Bertrand et al., 2010; Kissa and Herbomel, 2010; Sood et al., 2010; Bresciani et al., 2014; Gore et al., 2016; Tian et al., 2017). This region is also indicated as PBI. Due to the reduced blood flow and the presence of specific signals on the surface of HSPCs and endothelial cells of the caudal vein plexus (CVP), HSCPs leave the circulation and extravasate in the PBI region, where they are surrounded by endothelial and perivascular mesenchymal stromal cells that modulates their subsequent proliferative and/or differentiation fate (Murayama et al., 2006; Jin et al., 2007, 2009; Tamplin et al., 2015). The CHT region is the teleost homologous of the mammalian fetal liver and there, part of the HSCPs proliferates and gives rise to erythroid and myeloid cells, and some of them through circulation, move anteriorly to the thymus and to the anterior part of the kidney by 3 and 4 dpf, respectively (Kissa et al., 2008). Like in mammals, the zebrafish thymus is the site of differentiation and maturation of T-lymphocytes (Hess and Boehm, 2012), while the kidney marrow, where HSPCs reside through adulthood, is analogous to mammalian bone marrow (Bertrand and Traver, 2009). Both organs represent the sites where the adult hematopoiesis reside (de Jong and Zon, 2005).

## Zebrafish Models of Blood Disease

Historically, the zebrafish mutant *sauternes* (*sau*), represented the first example of a growing list of zebrafish contributions to the study of human diseases. Isolated during a large zebrafish forward genetic screening, the mutated gene encodes δ-aminolevulinate synthase (ALAS-2), an enzyme involved in the first step of heme biosynthesis (Brownlie et al., 1998). Notably, because mutations in ALAS2 gene are responsible for the congenital sideroblastic anemia (CSA) in humans, zebrafish *sauternes* mutants represented also the first animal model for the disease (Brownlie et al., 1998). Since then, through both forward and reverse genetic analysis, the zebrafish have proven to be instrumental in the study of human blood disorders (Berman et al., 2003; North and Zon, 2003; Forrester et al., 2012; Moore and Langenau, 2012; Santoriello and Zon, 2012; Zhang and Yeh, 2012; Avagyan and Zon, 2016; Robertson et al., 2016; Gore et al., 2018). In the following sections we will discuss a few of these disease models.

### Zebrafish Erythroid and Myeloid Models of Disease

#### Diamond–Blackfan Anemia

Diamond–Blackfan anemia (DBA) is a congenital bone marrow failure syndrome characterized by a complex array of hematopoietic and non-immunological defects. Patients with DBA are generally diagnosed during infancy or early childhood and present erythrocyte aplasia with anemia, macrocytosis, reticulocytopenia, and a paucity of red blood cell precursor cells within a normocellular marrow, associated with growth retardation, and limb, cardiac, and/or craniofacial malformations and have a predisposition to cancer (O’Brien et al., 2017). Other specific features of DBA are an elevated erythrocyte adenosine deaminase (ADA) activity and an elevated fetal hemoglobin concentration (McGowan and Mason, 2011). The primary treatment of DBA is corticosteroids, but ∼40% of patients lose steroid responsiveness, requiring chronic red cell transfusions (O’Brien et al., 2017). The only definitive treatment for DBA consists of hematopoietic stem cell transplantation (HSCT). In the 65% of patients DBA is caused by heterozygous mutations in ribosomal genes; while in the remaining ∼35% the genetics causes are still unknown (O’Brien et al., 2017). While, the most commonly mutated gene in DBA patients is RPS19 (Draptchinskaia et al., 1999; Campagnoli et al., 2008), germ-line mutations in genes encoding both small and large components of the ribosomal subunits (RPS24, RPS17, RPS7, RPS10, RPS26 and RPL35A, RPL5, RPL11, RPL26, respectively) have also been described (Farrar et al., 2011; Gazda et al., 2012).

In mice, homozygous knockout mutants for the rps19 gene presented early embryonic lethality (Matsson et al., 2004). Heterozygous mice did not present hematologic or developmental phenotypes, because of a genetic compensation from the wild-type rps19 locus (Matsson et al., 2006). Another mouse model with a ethylnitrosourea (ENU)-induced missense point mutation in rps19 gene showed a hematopoietic defect that was rescued by p53 knockdown (McGowan et al., 2008; McGowan and Mason, 2011).

To date two independent models of DBA were developed in zebrafish using MOs knockdown approaches against the ribosomal protein S19 (*rps19)* transcripts (Danilova et al., 2008; Uechi et al., 2008). In both cases, *rps19* knockdown recapitulated the phenotypes observed in DBA patients such as the defective erythropoiesis and morphologic abnormalities. A subsequent zebrafish knockdown model of *rpl11*, another ribosomal protein found mutated in DBA patients (Chakraborty et al., 2009), confirmed the central role for p53 activation in the pathophysiology of DBA (Ball, 2011). More recently, the genetic models of *rps19* and *rpl11* deficiency were developed (Zhang et al., 2014). Both models presented a reduction of protein production and in particular of globin proteins in red blood cells, suggesting that the protein reduction could be a key contributing factor to erythroid defects observed in DBA (Zhang et al., 2014). Finally, Danilova et al. (2018) investigated the role of immune system in DBA. Using *rpl11* mutants and *rps19* morphants, they showed an increased level of interferon network, inflammatory pathways and complement system suggesting that the activation of the innate immune system could contribute to the physiopathology of DBA (Danilova et al., 2018).

Recently, another gene of the rps family, rps29, has been associated with DBA using whole-exome sequencing and functional studies in a zebrafish model of rps29 deficiency (Taylor et al., 2012; Mirabello et al., 2014).

Finally, DBA patients present a high incidence of cancer, with particularly high risks of leukemia, osteosarcoma, myelodysplastic syndrome and colon adenocarcinoma (Vlachos et al., 2012); notably, *rps* and *rpl* heterozygous mutations also cause tumors in zebrafish (Amsterdam et al., 2004; Lai et al., 2009).

#### Erythropoietic Protoporphyria

The different inherited porphyrias are genetic diseases affecting heme biosynthesis, caused by mutations in specific enzymes of the heme biosynthetic pathway. As a result of these enzymatic deficiencies, the intermediates of the heme biosynthetic pathway (porphyrinogens, porphyrins and their precursors) are produced in excess and accumulate in tissues resulting in neurological, photo-cutaneous symptoms, and hematological disturbances (Richard et al., 2008). Based on which tissue accumulates porphyrin, this group of diseases can be divide into erythropoietic or hepatic. Three different erythropoietic porphyrias have been described: erythropoietic protoporphyria (EPP), which is the most frequent, congenital erythropoietic porphyria (CEP), and the very rare hepatoerythropoietic porphyria (HEP) (Richard et al., 2008). In humans, EPP is associated with inherited defects in the ferrochelatase (FECH) gene which catalyzes the insertion of a ferrous iron into protoporphyrin IX (PPIX) to form heme (Puy et al., 2010). The main clinical manifestation is painful skin inflammation after short exposure to sunlight. However, because the heme formation mainly occurs in the bone marrow and liver, mutations affecting FECH activity lead to PPIX accumulation in the bone marrow, erythrocytes, plasma, and liver and it has been estimated that up to 20% of the EPP patients have liver injury and approximately 2–5% develop serious liver damage or even liver failure (Wang et al., 2018).

After chemical mutagenesis using ENU, a viable autosomal recessive mutation in mouse fech gene was isolated and characterized in the early 1990s (Tutois et al., 1991; Boulechfar et al., 1993). Homozygous null mice present a severe reduction of FECH enzymatic activity and they exhibit jaundice, photosensitivity and dramatic hepatic dysfunction (Tutois et al., 1991).

A zebrafish genetic model of EPP, with mutations in the ferrochelatase (*fech*) gene, was obtained from a large-scale genetic screen (Childs et al., 2000). Zebrafish ferrochelatase mutants (*Dracula*) present a light-dependent lysis of red blood cells and liver disease (Childs et al., 2000).

A zebrafish mutant (*ype^*tp61*^*), which represented the first genetic model of HEP, was obtained in a forward genetic screen (Wang et al., 1998). The mutant presents a mutation in the *uroporphyrinogen decarboxylase* (*urod*) gene. Homozygous embryos die due to photo-ablation of their auto-fluorescent blood cells upon light exposure (Wang et al., 1998) and present clinical similarities to the defects observed in HEP patients.

#### Systemic Mastocytosis

Mastocytosis refers to a group of hematological disorders characterized by an increase in mast cell production, as well as abnormal morphology with aberrant surface receptor expression of tissue mast cells. These disorders are usually the result of various gain-of-function mutations affecting the tyrosine kinase KIT receptor, leading to increased accumulation and survival of tissue mast cells (Klaiber et al., 2017). Mastocytosis is divided in different subtypes: mastocytoma, urticaria pigmentosa, and systemic mastocytosis (SM), which represents the most severe subtype because mast cells accumulate in multiple organs. In the case of SM, a c-Kit D816V mutation is the most common cause of the disease and it gives rise to a constitutively active form of the protein that activates PI3K, Jak-STAT, and MAPK pathways (Lennartsson and Ronnstrand, 2012). Mouse models of SM using D816V mutation of human (Zappulla et al., 2005) or mouse (Gerbaulet et al., 2011) c-Kit gene have been developed. Similarly, a zebrafish transgenic model ubiquitously expressing the human KIT-D816V mutation has been developed (Balci et al., 2014). Adult transgenic fish demonstrate a myeloproliferative disease phenotype with a strong accumulation of mast cells in the kidney marrow and high expression levels of endopeptidases, consistent with SM defects observed in patients. Moreover, the zebrafish model showed a higher incidence of disease than in the transgenic mice overexpressing the same human mutant gene (Balci et al., 2014).

### Zebrafish Models of Primary Immunodeficiencies

Primary immunodeficiencies (PIDs) represent a heterogeneous group of genetic disorders characterized by the partial or complete absence of the immune system or its improper activity (Al-Herz et al., 2011). So far, more than 230 PID-causing genes have been identified and novel gene defects continue to be discovered (Al-Herz et al., 2011). Among PIDs, severe combined immunodeficiencies (SCIDs) are the most severe forms, resulting in a block of the development of T, B and/or NK cells and, consequently, in a high susceptibility to any kind of infection. For the most severe forms of PIDs the HSCT represents the current treatment of choice and, when a histocompatibility leukocyte antigen (HLA)-matched donor is not available, conditioning chemotherapy may be needed to facilitate robust and sustained engraftment of donor cells and improve immune reconstitution (Pai et al., 2014; Ott de Bruin et al., 2015). Some SCIDs have also been successfully treated with gene therapy (Fischer et al., 2013; Mukherjee and Thrasher, 2013). Unfortunately, for many cases of PIDs the genetic causes are still unknown or poorly understood (Shearer et al., 2014). Although current advances in analyzing the genome or exome sequences of patients and their relatives uncover many sequence polymorphisms (SNPs) possibly affecting the blood disorders, *in vivo* analysis still represent the golden standard to functionally confirm their effects and, from this point of view, the zebrafish can provide a good platform to test the functional consequences of different genetic variants (Iwanami, 2014).

#### Reticular Dysgenesis

Reticular dysgenesis is one of the most rare and severe forms of SCIDs. The disease is clinically characterized by congenital lymphopenia, lymphoid and thymic hypoplasia with agranulocytosis and sensorineural deafness (Hoenig et al., 2018) and is caused by mutations in adenylate kinase 2 (*ak2*) gene (Lagresle-Peyrou et al., 2009; Pannicke et al., 2009; Six et al., 2015). Ak2 protein is mostly located in the mitochondrial membrane space where it catalyzes the conversion of 1ATP + 1AMP = 2ADP sustaining the mitochondrial oxidative phosphorylation (Dzeja and Terzic, 2003). In mouse *ak2* mutations are embryonically lethal, therefore zebrafish represented an alternative to try to model the disease. The first attempts to model RD in zebrafish consisted of embryonic knockdown with a splicing-MO mimicking one of the mutations found in a patient (Pannicke et al., 2009). Overall, larvae showed a reduction of the *ikaros* signal in the thymus indicating an impairment of leukocyte development during definitive hematopoiesis (Pannicke et al., 2009). Recently, the first knockdown results were independently confirmed using two other different MOs and, more importantly, by two distinct zebrafish genetic models (with a missense point mutation and a frame shift mutation, respectively) and a patient-derived iPSCs model of RD (Rissone et al., 2015). As previously shown *in vitro* in fibroblast of RD patients, ak2-deficiency in zebrafish induces an increased level of oxidative stress resulting in increased apoptosis and cell death of the HSPC population. *In vitro* differentiated iPSCs recapitulate the promyeloid block of their differentiation that has been described in the bone marrow of RD patients (Lagresle-Peyrou et al., 2009; Hoenig et al., 2017). Notably, in zebrafish antioxidant treatments with *N*-acetyl cysteine or Glutathione (GSH) were able to reduce the cellular oxidative stress *in vivo* rescuing the hematopoietic phenotypes; moreover similar results were obtained in the RD-patient derived iPSCs model, where the GSH, but not the all-*trans* retinoic acid (ATRA), treatment was able to significantly increase the differentiation of AK2-deficient iPSCs into mature granulocytes (Rissone et al., 2015). Interestingly, a recent report showed that in ak2-deficiencient hematopoietic progenitors obtained from a different RD-patient derived iPSCs, the intracellular ATP redistribution is impaired with a strong ATP depletion in the nucleus and an altered global transcriptional profile (Oshima et al., 2018).

#### Wiskott–Aldrich Syndrome

The Wiskott–Aldrich syndrome (WAS) is a rare X-linked recessive disease (with an estimated incidence of less than 1 in 100,000 births) characterized by eczema, bleeding diathesis, and recurrent infections that occurs in boys (Ochs and Thrasher, 2006; Puck and Candotti, 2006). The disease is associated with mutations in a gene on the short arm of the X chromosome (Xp11.23) that was originally termed the WAS gene (Derry et al., 1994). The protein encoded by the WAS gene (WASp) is a major regulator of actin polymerization and it plays a role in the remodeling of the cytoskeleton during the formation of the immunological synapse between T cells and the and antigen-presenting cells. Mutations in WASp can prevent the formation of the immunologic synapse, impairing T-cell function and compromise the locomotion and the adhesion of other immunological cells such as B cells, macrophages, dendritic cells, etc. (Ochs and Thrasher, 2006; Puck and Candotti, 2006). Moreover, constitutively activating mutations of WASp are responsible for the X-linked severe congenital neutropenia (XLN) disorder (Devriendt et al., 2001).

Different knock-out and knock-in murine models of WAS were developed (Leon et al., 2016). The complete inactivation of WASp mimicked the thrombocytopenia although failed to reproduce the microcytosis observed in human patients (Sabri et al., 2006; Marathe et al., 2009). Notably, murine models have been successfully used to conduct preclinical trials evaluating somatic gene therapy as an alternative to transplantation (Dupre et al., 2006; Bosticardo et al., 2011, 2014; Uchiyama et al., 2012).

In zebrafish the *was* gene is duplicated and both present a very similar expression pattern (Cvejic et al., 2008). Morpholino analysis targeting *wasa* or *wasb* showed that they exhibit different levels of disruption to the wound inflammatory response. In particular, *wasa* morphants showed the strongest phenotypes, which consisted of impaired migration of neutrophils and macrophages in a tail wound assays and a thrombosis and/or bleeding phenotype that mirrored the human syndrome (Cvejic et al., 2008). Morpholino studies were then confirmed by two different mutant alleles (Cvejic et al., 2008). More recently, a zebrafish *wasa* null mutant allele modeling WAS and XLN disorders was characterized (Jones et al., 2013). The null mutant showed defects in the wound-induced inflammatory response, due to inefficiency in forming and maintaining new leading pseudopods, and also defects in immune-cell-mediated resistance to bacterial infection, as observed in WAS patients (Jones et al., 2013).

#### WHIM Syndrome

The warts, hypogammaglobulinemia, infections, and myelokathexis (WHIM) syndrome is caused by dominant mutations in chemokine receptor CXCR4 that induce the truncation of its carboxy-terminal domain. This leads to a defect in the internalization of the CXCR4 receptor after the binding to the sdf1 ligand (which is encoded in humans by CXCL12 gene) and it induces an increased signaling and enhanced migration after stimulation by chemokine (Hernandez et al., 2003). The WHIM syndrome is an inherited immunodeficiency that presents a range of symptoms, including human papillomavirus (HPV)-induced warts, reduced long-term immunoglobulin G (IgG) titers, recurring infections, retention of neutrophils in the bone marrow (myelokathexis), and leukopenia (Kallikourdis et al., 2015). In a mouse model of the WHIM syndrome, which recapitulates the defects observed in human patients, the expression of the mutant forms of CXCR4 in hematopoietic stem cells blocks the release of neutrophils from the bone marrow, inducing apoptosis in neutrophils and eventually neutropenia (Kawai et al., 2007). A stable transgenic line specifically expressing in neutrophils the homologous CXCR4 receptor truncation mutations found in WHIM patients was generated in zebrafish to model the disorder. As observed in the mouse model and in human patients, the zebrafish model showed neutrophil retention in hematopoietic tissue and an impairment of neutrophil motility and wound recruitment. The neutrophil retention is SDF1 dependent, because depletion of SDF1 using MOs restores neutrophil chemotaxis to wounds (Walters et al., 2010).

#### Chronic Granulomatous Disease

Chronic granulomatous disease (CGD) is an inherited PID caused by functional impairment of the NADPH oxidase complex in neutrophilic granulocytes and monocytes compromising their ability to produce ROS that are highly toxic to phagocytosed microorganisms. CGD is characterized by recurrent and severe infections, dysregulated inflammation, and autoimmunity, and patients are at increased risk of life-threatening infections with catalase-positive bacteria and fungi and inflammatory complications such as CGD colitis (Arnold and Heimall, 2017). Mutations in any of the five structural subunits of the NADPH oxidase complex result in defective ROS production that are highly toxic to phagocytosed microorganisms.

Although only mouse genetic models of CGD are currently available (Schaffer and Klein, 2013), studies in zebrafish using MOs targeting different components of the PHOX complex successfully demonstrated that phagocyte-mediated killing of *Candida albicans* (Brothers et al., 2011) and *Mycobacterium marinum* (Yang et al., 2012) are dependent on their ability to generate an oxidative burst (Harvie and Huttenlocher, 2015).

#### Leukocyte Adhesion Deficiency

Leukocyte Adhesion Deficiency (LAD) is a group of disorders characterized by devastating bacterial infections associated with an increased number of circulating neutrophils (neutrophilia) (Burns et al., 2017). So far, four different types of LAD have been described (Burns et al., 2017). The LADs are usually distinguished by Roman numerals, I, II, III, and IV. There are mouse models for each of the four diseases, and additional non-murine animal models for two of them (Hanna and Etzioni, 2012). In particular, LAD IV (also indicated as Rac2-deficiency), is a very rare autosomal recessive disorder in which loss of function Rac2 mutations cause defects of neutrophil F-actin assembly, adhesion and migration (Schaffer and Klein, 2013). Although, because of the additional role of RAC2 in the NADPH complex, the phenotypes of RAC2-deficiency overlap those observed in CGD. Due to RAC2’s role in cell adhesion and migration, and other pathways, the phenotypes are more severe than in CGD. Recently, two models of RAC2-deficiency have been developed in zebrafish (Deng et al., 2011) using MOs and expressing in zebrafish neutrophils the human dominant inhibitory Rac2D57N mutation found in patients, respectively. Both models present a failure in wound healing due to impaired neutrophil chemotaxis and in both, the neutrophils fail to respond to a *Pseudomonas aeruginosa* infection (Deng et al., 2011). As observed in patients, LAD fish exhibit neutrophilia from hematopoietic tissue without increased production of neutrophils and a defect in leaving the vasculature to reach the sites of tissue damage. Notably, neutrophil retention in the CHT of WHIM fish is reduced by the expression of Rac2D57N in neutrophils, suggesting that Rac2 signaling is also necessary in CXCR4-mediated neutrophil retention in hematopoietic tissues (Deng et al., 2011).

#### ZAP70 Deficiency

Zeta-chain (TCR) associated protein kinase, 70 kDa (ZAP70) deficiency is a rare form of SCID characterized by a deficit of mature CD8+ T cells along with a regular number of non-functional circulating CD4+ T cells unable to mount an effective T cell response (Arpaia et al., 1994; Chan et al., 1994; Elder et al., 1994; Hivroz and Fischer, 1994). ZAP70 is an important mediator of T cell activation, proliferation, and differentiation (Wang et al., 2010). Mouse models of the disease present an even more severe block in T cell differentiation phenotypes with a lack of mature T cells (Negishi et al., 1995; Kadlecek et al., 1998), that usually is explained by a compensatory effect induced in humans by spleen tyrosine kinase (SYK) protein (Kadlecek et al., 1998). Although defects in lymphatic or blood endothelial specification have not been reported for ZAP70-deficient mice or humans, a first model in zebrafish using knockdown approaches indicated a role for both *syk* and *zap70* in vascular embryonic development (Christie et al., 2010). However, a more recent zebrafish genetic model, where a frame-shift mutation was introduced using a TALEN targeting exon 2 of the *zap70* gene, failed to show any vascular and/or lymphatic defects (Moore et al., 2016). However, zebrafish *zap70* null mutants presented a reduction of developing thymocytes and mature T cells during embryo development and later they develop a T cell-specific immunodeficiency that cannot be compensated by syk protein, fully confirming the data obtained in mouse models (Moore et al., 2016).

## Conclusion and Future Perspectives

Thanks to the next-generation sequencing techniques, the ability to identify gene defects in small populations or even in single patients with inherited diseases is increased rapidly. More importantly, the overall costs of whole-exome sequencing and whole genome-wide association studies are steadily dropping and, based on all the predictions, they will continue to decrease in the coming years. In this scenario, the use of the latest genome-editing techniques such as CRIPR/Cas9 in association with the numerous unique features of the zebrafish model, will represents a huge boost in the modeling and in the understanding of the physiopathology of human diseases.

## Author Contributions

All authors listed have made a substantial, direct and intellectual contribution to the work, and approved it for publication.

## Conflict of Interest Statement

The authors declare that the research was conducted in the absence of any commercial or financial relationships that could be construed as a potential conflict of interest.
